# Left ventricular strain derived from cardiac magnetic resonance can predict outcomes of pulmonary valve replacement in patients with repaired tetralogy of Fallot

**DOI:** 10.3389/fcvm.2022.917026

**Published:** 2022-08-18

**Authors:** Baiyan Zhuang, Shiqin Yu, Zicong Feng, Fengpu He, Yong Jiang, Shihua Zhao, Minjie Lu, Shoujun Li

**Affiliations:** ^1^Department of Magnetic Resonance Imaging, Cardiovascular Imaging and Intervention Center, Fuwai Hospital, State Key Laboratory of Cardiovascular Disease, National Center for Cardiovascular Diseases, Chinese Academy of Medical Sciences and Peking Union Medical College, Beijing, China; ^2^Pediatric Cardiac Surgery Center, Fuwai Hospital, State Key Laboratory of Cardiovascular Disease, National Center for Cardiovascular Diseases, Chinese Academy of Medical Sciences and Peking Union Medical College, Beijing, China; ^3^Department of Cardiovascular Surgery, The First Affiliated Hospital, Zhejiang University College of Medicine, Hangzhou, China; ^4^Department of Echocardiography, Fuwai Hospital, State Key Laboratory of Cardiovascular Disease, National Center for Cardiovascular Diseases, Chinese Academy of Medical Sciences and Peking Union Medical College, Beijing, China; ^5^Department of Echocardiography, Fuwai Hospital Chinese Academy of Medical Sciences, Shenzhen, China; ^6^Key Laboratory of Cardiovascular Imaging (Cultivation), Chinese Academy of Medical Sciences, Beijing, China

**Keywords:** strain rate, strain, cardiac magnetic resonance imaging, pulmonary valve replacement, repaired tetralogy of Fallot

## Abstract

**Purpose:**

Several adults with repaired tetralogy of Fallot (rToF) undergo pulmonary valve replacement (PVR) to reduce the right ventricular volume and retain right ventricular function. However, there is currently no consensus on the ideal time for PVR surgery in asymptomatic patients with rTOF with pulmonary regurgitation (PR). Clinical outcomes after PVR are also indeterminate. Recently, myocardial strain and strain rate derived from cardiac magnetic resonance (CMR) feature tracking were found to be more sensitive to right ventricular dysfunction than conventional parameters and therefore may add prognostic value in patients with rToF. We aimed to analyze whether pre-PVR left ventricular (LV) strain and strain rate detected by CMR feature tracking are associated with midterm outcomes after PVR in patients with rToF.

**Methods:**

Seventy-eight asymptomatic patients with rToF who required PVR due to moderate or severe PR were prospectively enrolled between January 2014 and June 2020. CMR cine sequences were obtained, and feature tracking parameters were measured preoperatively. Adverse events were documented during the follow-up. Receiver operating characteristic analysis was performed to determine the cutoff value. Kaplan–Meier curves were drawn with log-rank statistics; moreover, univariate and multivariate Cox proportional hazards regression analyses and Harrel C-indices were analyzed.

**Results:**

During 3.6 ± 1.8 years of follow-up, 25 adverse events were recorded. Kaplan–Meier survival curves and univariate Cox analysis verified that patients with significantly reduced radial strain (RS), circumferential strain (CS), longitudinal strain (LS), RS rate at systole and diastole (RSRs and RSRe), and circumferential and LS rates at diastole (CSRe and LSRe) had worse event-free survival. After multivariate correction, only LS and LSRe remained significantly associated with adverse outcomes (hazard ratio = 1.243 [1.083–1.428] and 0.067 [0.017–0.258], respectively, all *p* < 0.05). The cutoff values of LS and LSRe were −12.30 (%) and 1.07 (s^–1^), respectively.

**Conclusion:**

The LV strain and strain rate prior to PVR are important prognostic factors for adverse events after PVR in rToF.

## Introduction

Tetralogy of Fallot (ToF) is one of the most common types of congenital heart disease, affecting 356 per million live births (1). When surgical repair of ToF (rToF) is performed in early childhood, the natural history of the disease dramatically changes and survival in adulthood improves. However, pulmonary regurgitation (PR) usually occurs after rToF, leading to poor prognosis. There is 40–85% of patients who develop moderate to severe PR in 5–10 years after repair (2, 3). Pulmonary valve replacement (PVR) was introduced to reduce late complications, prior symptoms, and worsening function (4). Although an increasing number of studies have reported PVR as the treatment of choice for severe PR after rToF, the effects of PVR on ventricular remodeling remain controversial.

The guidelines issued by the AHA/ACC and ESC recommend the right and left ventricular end-diastolic volume index (RVEDVI and LVEDVI), tricuspid regurgitation (TR), arrhythmia, and other cardiac function and clinical parameters to determine the timing of PVR surgery (4, 5). The AHA/ACC recommends that adults with previous ToF and severe PR undergo PVR surgery when they have moderate to severe RV dysfunction or enlargement. ESC experts believed that normalization of RV size after reintervention is unlikely as soon as the end-systolic index exceeds 80 ml/m^2^ and the end-diastolic volume index exceeds 160 ml/m^2^; however, this cutoff for reintervention may not correlate with clinical benefit. A more sensitive parameter that can predict adverse outcomes at an earlier stage may help identify the optimal time for PVR surgery (6).

In comparison with the traditional measurements, including ejection function (EF) and ventricular volume, the myocardial strain detected by cardiac magnetic resonance (CMR) feature tracking, which reflects myocardial deformation, has added value to predict the outcome of various cardiovascular diseases (7, 8). Although without impaired EF, myocardial strain could still be a sensitive parameter for evaluating ventricular dysfunction and may add clinical value during follow-up (9).

Owing to ventricular coupling, LV strain is also implicated in RV volume overload (10). Thus, LV strain has the potential to provide valuable information prior to PVR. However, there is little data and no consensus on the use of CMR feature tracking to help determine the timing of surgical intervention for PR in patients with rToF (6).

To date, there have been studies comparing the changes in strain and strain rate before and after PVR (11–13) or the relationship between strain and ventricular function parameters (5, 14, 15); however, few of these studies analyzed the relationship between strain and post-PVR surgical outcomes. We aimed to comprehensively evaluate the predictive value of LV strain and strain rate using a custom feature-tracking algorithm applied to a conventional CMR cine in asymptomatic patients with rToF and PVR. We assumed that LV strain and strain rate could have a potential predictive value.

## Materials and methods

### Patient enrolment

This prospective study was conducted at our hospital. The study protocol was approved by the ethics committee of our hospital. All participants provided written informed consent prior to enrolment.

### Data definition

Asymptomatic patients were defined as those who maintained a functional status in New York Heart Association class I or II without arrhythmia or heart failure symptoms/signs that cause syncope (16).

The severity of PR was divided into absent, trivial, mild, moderate, and severe by transthoracic echocardiography (16). Patients underwent follow-up through either a clinic or *via* telephone inquiring with patients or their contacts every 3 months after enrolment. Adverse events that occurred after PVR surgery were recorded. Adverse events included sudden cardiac death (unexpected death either within 1 h of the onset of cardiac symptoms without progressive cardiac deterioration, when asleep, or within 24 h of last being seen alive) (17), cardiac transplantation, application of implantable cardioverter defibrillator (ICD) (18), heart failure (19), and arrhythmia-induced syncope (“syncope caused by arrhythmias” was adjudicated based on clinical symptom and ECG monitor). The syncope cases were defined position-independent, with few prodromal symptoms, and may occur with cyanosis, dyspnea, arrhythmias, weak heart sounds, and associated ECG-detected arrhythmias) (20, 21), reoperation for PVR, sustained atrial or ventricular arrhythmias analyzed by 24-h Holter monitoring (last ≥ 30s but not causing syncope), and cardiac catheterization ablation (22).

### Inclusion and exclusion criteria

Patients with rToF who were candidates for PVR were initially screened for this study from January 2014 to June 2020. According to the indications for PVR in asymptomatic patients with moderate or severe PR after rToF recommended by Boston Children’s Hospital in 2013 (22), asymptomatic patients with moderate to severe pulmonary valve regurgitation after rToF (regurgitation fraction by CMR ≥ 25%) meeting at least two of the following conditions were included: (1) RVEDVI > 150 ml/m^2^, (2) RV end-systolic volume index (RVESVI) > 80 ml/m^2^, (3) RVEF < 47%, (4) LVEF < 55%, (5) QRS duration > 160 ms, (6) sustained tachyarrhythmia related to right heart volume load, and (7) the presence of other hemodynamically significant lesions.

The following exclusion criteria were established: (1) obvious symptoms, (2) the presence of residual severe right ventricle outflow tract obstruction (RVOTO; ECG calculated gradient ≥ 60 mmHg), (3) RV pressure surpassing or equal to LV pressure, (4) ICD insertion or history of PVR, (5) contraindications for CMR or surgery, and (6) incomplete or poor-quality CMR (9).

### Standard imaging protocol

CMR examinations were performed on a 3.0 T system within 6 months prior to PVR surgery using a 3.0 T scanner (Ingenia, Philips Healthcare, Best, Netherlands) with front and back surface coils and retrospective ECG triggering for capture of the entire cardiac cycle by the same senior operator. Cine images were obtained using a balanced steady-state free precession (SSFP) sequence, including 2-chamber, 4-chamber, and short-axis acquisitions with 25 image frames per cardiac cycle. The short-axis scans covered the entire LV (nine slices; the following standard acquisition parameters used for routine clinical imaging were applied: field of view, 321 × 321 mm^2^; matrix, 184 × 256; slice thickness, 6 mm; TR/TE/flip-angle: 3 ms/1.6 ms/45°; temporal resolution, 43 ms; and parallel acquisition technique factor, 2). The two-dimensional phase-contrast images perpendicular to the main pulmonary artery (PA) were used for quantification of the PR fraction (field of view, 350 × 321 mm^2^; matrix, 140 × 123; slice thickness, 6 mm; TR/TE/flip-angle: 4.9 ms/2.9 ms/10°; parallel acquisition technique factor, 1).

### Surgery

All PVR surgeries were completed under cardiopulmonary bypass with mild hypothermia or a beating heart (without right-to-left shunt) by the same surgeon. A longitudinal incision was made in the pulmonary artery trunk or the position where the patch was previously placed. The replacement valve was inserted in the orthotropic position.

### Data analysis

The LV and RV cardiac function analysis and strain analysis were measured using CVI 42 software (Circle Cardiovascular Imaging Inc., Calgary, Canada) with the help of artificial intelligence (with more than 3 years of work experience and more than 600 cases of cardiac MRI analysis) (23). The endocardial and epicardial borders of the LV were semi-automatically defined using the AI function of the CVI following manual adjustments. Measurements included RA and LA volumes, diastolic and systolic RV and LV volumes, mass, ventricular stroke volumes, and EFs. The ventricular volumes and mass were indexed by body surface area (BSA) into RA volume index (RAVI), LA volume index (LAVI), LVEDVI, LV end-systolic volume index (LVESVI), indexed RVEDVI, and RVESVI. Heart rate and QRS duration were obtained using electrocardiography. The PR fraction was calculated from the CMR flow velocity mapping. The severity of RVOTO was reflected by the pulmonary artery systolic gradient from the ECG. RV mass/volume ratio was calculated to reflect the degree of right ventricular hypertrophy (RVH). The RV pressure was measured using TR.

**FIGURE 1:**
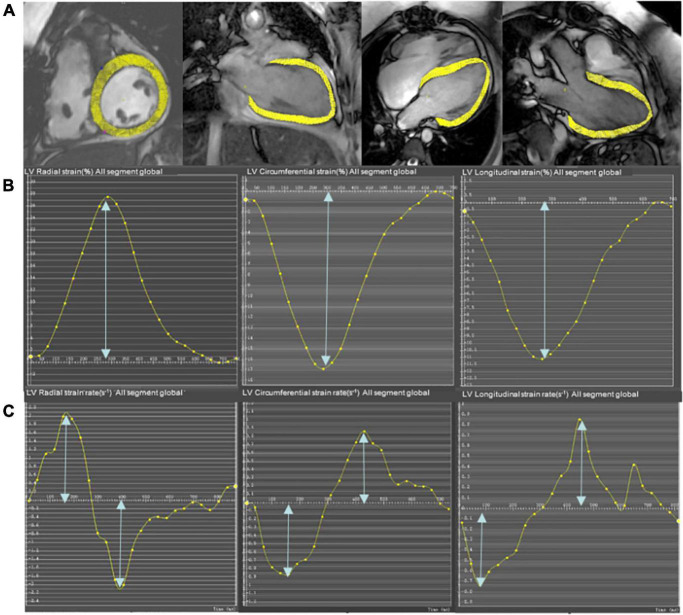
Representative short-axis and long-axis images **(A)**, strain curves **(B)**, and strain rate curves **(C)** in a participant who underwent pulmonary valve replacement (PVR). The epicardial and endocardial borders (excluding papillary muscles and trabeculae) at end-diastole are semi-manually defined in the LV 4-chamber, LV 2-chamber, and LV 3-chamber to calculate longitudinal strain and strain rate. The entire LV short-axis SSFP cine images are used to calculate radial and circumferential strain and strain rate.

The global cardiac strain and strain rate representing the average of the entire heart were calculated from CMR cine images using the mature feature tracking software CVI 42 version 5.12. The method has been described in detail in a previous study (15). In brief, the LV 4-chamber, LV 3-chamber, and LV 2-chamber were selected for post-processing to calculate the peak longitudinal strain (LS), peak systolic strain rate, and peak early diastolic strain rate (LSRe). The entire LV short-axis SSFP cine images were analyzed for radial strain (RS), circumferential strain (CS), peak systolic strain rate (RSRs and CSRs), and early diastolic strain rate (RSRe and CSRe). The endocardial and epicardial borders of the LV were semi-automatically defined using the AI function of the CVI following manual adjustments. The peak radial, circumferential, and LSs of the LV were identified as the highest or lowest peak of the global strain curve. Similarly, the peak systolic and early diastolic strain rates of the LV were calculated as the highest or lowest peak of the global strain rate curve (Figure 1) (24).

### Inter- and intra-reproducibility

Intra- and inter-observer reliabilities were assessed in 20 randomly selected patients using Bland–Altman plots. Intra-observer reliability was derived from repeated measurements by one radiologist (^**^) after at least 1 week of blinding to previous results. Inter-observer reliability was independently assessed by two radiologists with more than 3 years of work experience and more than 650 cases of cardiac MRI analysis (^**^ and AA), where one radiologist measured once and then a second radiologist measured again (blinded to the first radiologist’s measurements).

### Statistics

Statistical analyses were performed using SPSS Statistics (version 22.0; IBM Corp., Armonk, NY, United States) and MedCalc (version 18.2.1, Ostend, Belgium). Continuous data were descriptively reviewed and statistically analyzed using the Kolmogorov–Smirnov test to check for normality of the continuous variables. Continuous variables were described as means ± standard deviations or as median values with interquartile range, with respect to the normality of distribution. Comparisons between demographic and strain variables determined in the adverse event and non-adverse event groups were performed using either Student’s *t*-test or Wilcoxon rank-sum test on the basis of whether the data were normally distributed (24). The Bonferroni method was used to correct multiple comparisons. Categorical variables were summarized by frequencies and percentages and compared between patients with and without the outcomes using the chi-squared test or Fisher’s exact test.

To visualize the difference in event-free survival between different patient categories, Kaplan–Meier curves were drawn, followed by the log-rank test. The optimal preoperative strain and strain rate cutoffs were identified using receiver operating characteristic (ROC) curve analysis using the Youden index. The ROC curve was then drawn with the corresponding calculated area under the curve (AUC). The parameters satisfying the pH assumption were included in the univariate Cox regression analysis. Univariate Cox regression analysis was used to evaluate the predictive value of the strain and strain rate. Multivariate analysis Cox regression (forward LR) was performed for those parameters that were confirmed to be statistically significant in univariate analysis (*P* < 0.05) to identify the independent risk factors of prospective adverse events. Harrel C-indices were used to compare the relative predictive abilities of strain and strain rate. Statistical significance was set at *P* < 0.05.

## Results

### Characteristics of study participants

**FIGURE 2:**
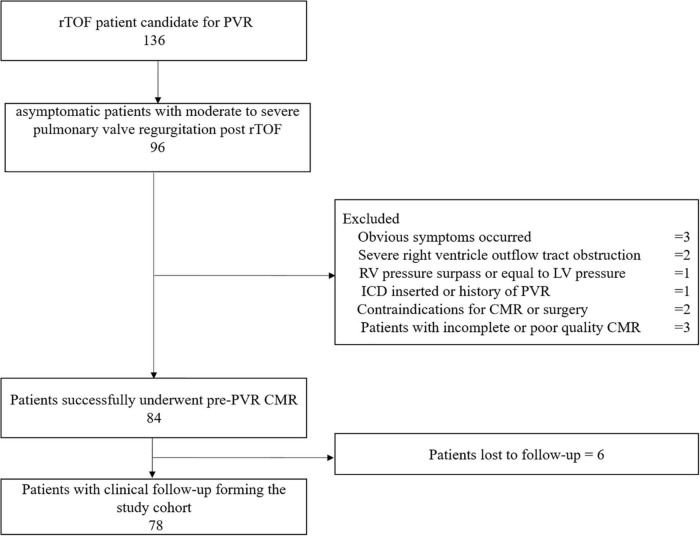
This flow chart shows the number of patients at each stage of the study. PVR, pulmonary valve replacement; ICD, implantable cardioverter defibrillator; ToF, Tetralogy of Fallot; CMR, cardiac magnetic resonance.

From January 2014 to June 2020, 78 asymptomatic patients with rToF with moderate or severe PR (21.5 years old, 44% male) were included for analysis with an average follow-up duration of 3.6 ± 1.8 years. The reasons for failure to be included are detailed in the study flowchart (Figure 2). The patients involved in our study did not have coronary artery anatomy abnormalities; only two patients had aortic arch abnormalities (patent ductus arteriosus). Demographic, surgical, ECG, and CMR parameters are summarized in Table 1. If appropriate, the parameters were indexed to BSA. On average, the age of the patients who underwent primary rToF was 3 (interquartile range [IQR]: 0–9) years. The most common approach was the transannular patch (*n* = 56). The mean QRS duration (150.78 ± 30.89 ms) was extended. On average, patients had slightly reduced LVEF (50.47 ± 10.94%) and RVEF (38.37 ± 12.53%). Meanwhile, the RVEDVI and RVESVI were significantly enlarged (168.8 ± 54.36 and 104.42 ± 43.06 ml/m^2^, respectively). The mean PR fraction was 49.98 ± 18.58%.

**TABLE 1:** Baseline demographic, electrocardiographic and CMR parameters of the study subjects (*n* = 78).

Variable		
Male		44%
Height (cm)		1.53 ± 0.5
Weight (kg)		55.57 ± 15.52
Body surface area (m^2^)		1.57 ± 0.27
Age at surgical repair (years)		3 (0–9)
Age at PVR (years)		21.5 (14–32)
Age at CMR (years)		21.5 (14–32)
Cardiac symptoms prior to PVR		63 (80.7%)
Transannular patch		56 (71.7%)
NYHA Class I or II prior to PVR		65 (83.3%)
NYHA Class III or IV prior to PVR		14 (17.9%)
Prescribed cardiac medications prior to PVR		9 (11.5%)
≥3previous cardiac operations		9 (11.5%)
Related familial genetic syndromes		5 (6.4%)
**Valve types**		
	Bioprosthesis	47 (60.2%)
	Homograft	12 (15.3%)
	mechanical valve	30 (38.5%)
**Electrocardiogram**		
	QRS duration (ms)	150.78 ± 30.89
	Heart rate (beats/min)	76.68 ± 14.55
	Sustained atrial arrhythmias prior to PVR	12 (15.3%)
	Sustained ventricular arrhythmias prior to PVR	0
**Echocardiography**		
	Tricuspid systolic pressure gradient (mmHg)	31.4 (25.5, 43.2)
	Tricuspid diastolic pressure gradient (mmHg)	2 (1.4, 3.2)
	Tricuspid systolic velocity (m/s)	2.8 (2.6, 3.275)
	Tricuspid diastolic velocity (m/s)	0.7 (0.6, 0.9)
	≤Mild tricuspid regurgitation	39 (50%)
	≥Moderate tricuspid regurgitation	34 (43.6%)
	Pulmonary valve systolic pressure gradient (mmHg)	15.2 (7.8, 25.5)
	Pulmonary valve diastolic pressure gradient (mmHg)	19.97 ± 8.6
	Pulmonary valve systolic velocity (m/s)	2.09 ± 0.72
	Pulmonary valve diastolic velocity (m/s)	2.3 (2, 3.15)
	≤Mild pulmonary valve regurgitation	0
	≥Moderate pulmonary valve regurgitation	78 (100%)
	Aortic valve systolic pressure gradient (mmHg)	3.6 (2.6, 4.8)
	Aortic valve diastolic pressure gradient (mmHg)	1 (0.875, 1.2)
	Aortic valve systolic velocity (m/s)	0.95 ± 0.25
	Aortic valve diastolic velocity (m/s)	0.9 (0.7, 1.8)
	≤Mild aortic valve regurgitation	14 (17.9%)
	≥Moderate aortic valve regurgitation	2 (2.6%)
**CMR parameters pre-PVR**		
	Pulmonary regurgitation fraction (%)	49.98 ± 18.58
	LV end-diastolic volume (ml)	142.01 ± 54.00
	LV end-systolic volume (ml)	75.51 ± 44.60
	LV ejection fraction (%)	50.47 ± 10.94
	LV mass-ED (g)	67.60 ± 26.64
	LVEDVI (ml/m^2^)	89.98 ± 25.86
	LVESVI (ml/m^2^)	47.48 ± 22.71
	RV end-diastolic volume (ml)	263.56 ± 91.42
	RV end-systolic volume (ml)	164.07 ± 72.53
	RV ejection fraction (%)	38.37 ± 12.53
	RVEDVI (ml/m^2^)	168.80 ± 54.36
	RVESVI (ml/m^2^)	104.42 ± 43.06
	RV mass-ED (g)	64.28 ± 30.22
	RV mass index (g/m^2^)	41.12 ± 19.74
	RV mass/RV end-diastolic volume ratio (g/ml)	0.40 ± 0.20
	RV/LV end-diastolic volume ratio (g/ml)	2.00 ± 0.74
**Additional procedures with PVR**		
	Tricuspid repair	26 (33.3%)
	Patent ductus arteriosus cut and suture	2 (2.5%)
	Ventricular septal defect closure	13 (16.7%)
	Atrial septal defect closure	3 (3.8%)
	Pulmonary angioplasty	11 (14.1%)
	Right ventricular outflow tract muscle resection	7 (8.9%)
	Aortic valve replacement	5 (6.4%)
	Prior aortopulmonary shunt	1 (1.3%)
	Subaortic septum resection	2 (2.7%)

PVR, Pulmonary valve replacement; CMR, cardiac magnetic resonance imaging; LV, left ventricle; RV, right ventricular; LVEDVI, indexed LV end-diastolic volume; LVESVI, indexed LV end-systolic volume; RVEDVI, indexed RV end-diastolic volume; RVESVI, indexed RV end-systolic volume.

### Outcomes and cardiac magnetic resonance parameters

**FIGURE 3:**
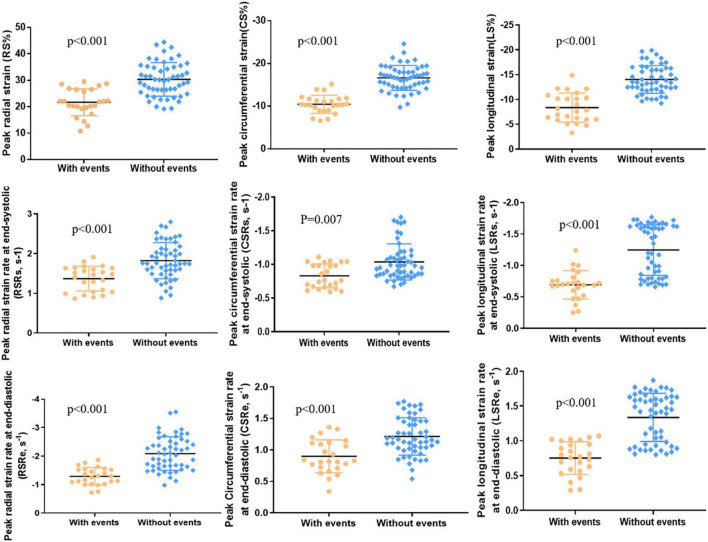
LV peak radial strain (RS), peak circumferential strain (CS), peak longitudinal strain (LS), peak radial strain rate at end-systole (RSRs), peak circumferential strain rate at end-systole (CSRs), peak longitudinal strain rate at end-systole (LSRs), peak radial strain rate at end-diastole (RSRe), peak circumferential strain rate at end-diastole (CSRe), and peak longitudinal strain rate at end-diastole (LSRe) in patients with adverse events (*n* = 25) and patients without adverse events (*n* = 53).

A total of 25 (32%) patients had noted adverse events (cardiovascular death, 3; ICD for ventricular tachycardia combined with atrioventricular block, 1; heart failure, 9; syncope caused by arrhythmias, 6; sustained atrial or ventricular arrhythmias, 4; and cardiac catheterization ablation for atrial fibrillation, 2), and 53 (68%) patients did not have any adverse events. Significant differences were noted between the two groups in LV RS, CS, and LS (all *p* < 0.001; Table 2 and Figure 3). The values in patients with adverse events were lower. Similarly, patients with adverse events noted tended to have lower absolute preoperative LV RSRs (1.37 ± 0.31 vs. 1.88 ± 0.59 s^–1^, *p* < 0.001) and RSRe (−1.2 ± 0.57 vs. −2.28 ± 0.93 s^–1^, *p* < 0.001). Furthermore, patients with adverse events noted had lower absolute preoperative CSRe (0.90 ± 0.26 vs. 1.23 ± 0.33 s^–1^, *p* < 0.001) and LSRe (0.79 ± 0.31 vs. 1.34 ± 0.35 s^–1^, *p* < 0.001) than those without adverse events. Significant differences were also observed in heart rate, age at surgical repair, age at PVR, LVEF, LVESVI, RVESVI, RV mass, and RV mass index between the two groups (Table 2). However, the QRS duration and PR fraction between the two groups did not exhibit a significant difference.

**TABLE 2:** Comparison of baseline parameters and LV strain parameters (mean ± SD/median and interquartile range) between patients with or without adverse events.

Variable	Event (*n* = 25)	No event (*n* = 53)	*P-value*
Male	46%	44%	0.385
Age (years)	22.81 ± 10.62	29.12 ± 13.37	0.069
Height (cm)	1.62 ± 0.15	1.62 ± 0.13	0.730
Weight (kg)	55.08 ± 15.34	56.56 ± 16.13	0.648
Body surface area (m^2^)	1.57 ± 0.28	1.58 ± 0.25	0.615
Age at surgical repair (years)	9 (3, 16)	2 (0, 7)	0.002
Age at PVR (years)	31 (16, 43)	20 (14, 28)	0.025
Age at CMR (years)	30 (15, 43)	20 (14, 27)	0.035
Predicted RV pressure by TR, mmHg	57.27 ± 14.95	53.74 ± 9.66	0.290
QRS duration (ms)	147.84 ± 31.1	156.56 ± 30.27	0.438
Heart rate (bpm)	74.5 ± 12.2	81.04 ± 17.86	0.034
Mean pulmonary valve area (mm^2^)	9.01 ± 3.65	7.68 ± 3.59	0.043
Pulmonary regurgitation fraction (%)	52.95 ± 18.5	48.64 ± 18.65	0.645
Predicted RV pressure by TR (mmHg)	57.27 ± 14.95	53.74 ± 9.66	0.560
LV LGE	5 (24%)	2 (3.77%)	0.012
LAVI (ml/m^2^)	47.6 (31.4, 68.0)	29.7 (23.2, 38.9)	<0.001
LVEDV (ml)	135.69 ± 50.7	154.65 ± 59.05	0.042
LVESV (ml)	69.65 ± 43.96	87.23 ± 44.38	0.020
LV ejection fraction (%)	53.21 ± 10.66	44.99 ± 9.51	0.001
LV mass at end-diastole (g)	65.49 ± 27.2	71.8 ± 25.49	0.152
LVEDVI (ml/m^2^)	86.49 ± 21.04	96.97 ± 32.87	0.122
LVESVI (ml/m^2^)	43.87 ± 20.5	54.7 ± 25.49	0.035
LV mass index (g/m^2^)	44.81 ± 13.26	41.66 ± 12.76	0.186
LV mass/volume (g/ml)	0.5 ± 0.18	0.5 ± 0.15	0.868
RV LGE	1 (4%)	1 (1.89%)	0.547
RAVI (ml/m^2^)	57.6 (35.4, 78.0)	39.7 (21.2, 52.9)	<0.001
RVEDV (ml)	258.99 ± 82.16	272.54 ± 108.54	0.609
RVESV (ml)	158.62 ± 66.33	174.75 ± 83.76	0.453
RV ejection fraction (%)	39.97 ± 12.19	35.24 ± 12.84	0.058
RVEDVI (ml/m^2^)	165.76 ± 45.12	174.77 ± 69.69	0.500
RVESVI (ml/m^2^)	100.21 ± 36.59	112.68 ± 53.4	0.034
RV mass (g)	68.59 ± 26.78	59.97 ± 33.66	0.045
RV mass index (g/m^2^)	43.54 ± 15.94	38.69 ± 23.54	0.024
RV mass/RV end-diastolic volume ratio (g/ml)	0.27 ± 0.1	0.24 ± 0.14	0.053
RV/LV end-diastolic volume ratio (g/ml)	1.95 ± 0.75	2.04 ± 0.66	0.728
RS (%)	21.67 ± 5.06	31.85 ± 8.9	<0.001
CS (%)	−10.46 ± 2.11	−16.64 ± 2.91	<0.001
LS (%)	−8.36 ± 2.93	−14.04 ± 2.79	<0.001
RSRs (s^–1^)	1.37 ± 0.31	1.88 ± 0.59	<0.001
CSRs (s^–1^)	−0.76 (−0.66, −1.00)	−0.98 (−0.84, −1.14)	0.007
LSRs (s^–1^)	−0.66 ± 0.31	−1.35 (−0.79, −1.62)	<0.001
RSRe (s^–1^)	−1.2 ± 0.57	−2.28 ± 0.93	<0.001
CSRe (s^–1^)	0.90 ± 0.26	1.23 ± 0.33	<0.001
LSRe (s^–1^)	0.79 ± 0.31	1.44 (0.94, 1.63)	<0.001

PVR, Pulmonary valve replacement; CMR, cardiac magnetic resonance imaging; LV, left ventricle; RV, right ventricular; LVEDVI, indexed LV end-diastolic volume; LVESVI, indexed LV end-systolic volume; RVEDVI, indexed RV end-diastolic volume; RVESVI, indexed RV end-systolic volume; LGE, late gadolinium enhancement.

### Predictors for adverse events

**FIGURE 4:**
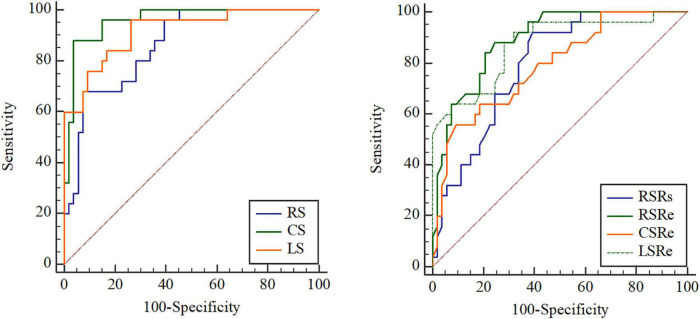
Receiver operating characteristic (ROC) curve analysis of the prognostic performance of peak radial strain (RS), peak circumferential strain (CS), peak longitudinal strain (LS), peak systole radial strain rate (RSRs), peak early diastole radial strain rate (RSRe), peak circumferential strain rate at end-diastole (CSRe), and peak early diastole longitudinal strain rate (LSRe) in asymptomatic patients with repaired tetralogy of Fallot (rToF) who required pulmonary valve replacement (PVR) for moderate or severe pulmonary regurgitation.

After ROC analysis, the corresponding cutoff values, sensitivity, specificity, and AUC of clinical parameters, different preoperative function parameters, strains, and strain rates for predicting adverse outcomes were obtained (Table 3). We found that the AUCs of the strain parameters were the best among the abovementioned parameters. The cutoff values of RS, CS, and LS were 22.2% (68.00% sensitivity, 92.45% specificity), −12.35% (88.00% sensitivity, 96.23% specificity), and −12.3% (96.00% sensitivity, 73.58% specificity), respectively. With regard to strain rates, the cutoff values of RSRs, RSRe, CSRe, and LSRe were 1.69 s^–1^ (92.00% sensitivity, 60.38% specificity), −1.65 s^–1^ (88.00% sensitivity, 75.47% specificity), 0.84 s^–1^ (56.00% sensitivity, 90.57% specificity), and 1.07 s^–1^ (92.00% sensitivity, 67.92% specificity), respectively. The AUCs of RS, CS, LS, RSRs, RSRe, CSRe, and LSRe were 0.863, 0.959, 0.916, 0.792, 0.889, 0.788, and 0.871, respectively (Figure 4 and Table 3).

**TABLE 3:** Receiver operating characteristic (ROC) curve analysis.

Variable	AUC	Youden index J	Associated criterion	Sensitivity (%)	Specificity (%)	*P-value*
RS (%)	0.863 (0.766–0.93)	0.6045	≤22.20	68.00	92.45	<0.001
CS (%)	0.959 (0.888–0.99)	0.8423	>−12.35	88.00	96.23	<0.001
LS (%)	0.916 (0.831–0.96)	0.6958	>−12.30	96.00	73.58	<0.001
RSRs (s^–1^)	0.792 (0.685–0.87)	0.5238	≤1.69	92.00	60.38	<0.001
CSRs (s^–1^)	0.873 (0.779–0.93)	0.5902	>−0.78	76.00	83.02	<0.001
LSRs (s^–1^)	0.691 (0.576–0.79)	0.3857	>−0.79	48.00	90.57	<0.001
RSRe (s^–1^)	0.889 (0.798–0.94)	0.6347	>−1.65	88.00	75.47	<0.001
CSRe (s^–1^)	0.788 (0.681–0.87)	0.4657	≤0.84	56.00	90.57	<0.001
LSRe (s^–1^)	0.871 (0.775–0.93)	0.5992	≤1.07	92.00	67.92	<0.001
LVEDVI (ml/m^2^)	0.596 (0.455–0.737)	0.2113	>97.48	40.00	81.13	0.181
LVESVI (ml/m^2^)	0.635 (0.497–0.774)	0.2913	>51.07	48.00	81.13	0.055
RVEDVI (ml/m^2^)	0.548 (0.403–0.693)	0.1223	>190.33	36.00	76.23	0.519
RVESVI (ml/m^2^)	0.638 (0.498–0.778)	0.2226	>108.42	60.00	62.26	0.053
LV mass-ED (g)	0.58 (0.397–0.764)	0.1849	>62.00	60.00	58.49	0.374
LV mass/volume (g/ml)	0.475 (0.287–0.662)	0.1592	>0.44	48.00	67.92	0.778
RV mass-ED (g)	0.716 (0.558–0.873)	0.3358	>63.11	60.00	73.58	0.017
RV mass index (g/m^2^)	0.763 (0.619–0.906)	0.4015	>35.81	76.00	64.15	0.004
RV mass/volume (g/ml)	0.743 (0.597–0.89)	0.2596	>0.18	92.00	33.96	0.007
RV/LV end-diastolic volume ratio	0.506 (0.317–0.695)	0.1623	≤1.04	20.00	96.23	0.948
Predicted RV pressure by TR (mmHg)	0.495 (0.326–0.664)	0.1693	>48.36	73.68	43.24	0.957
QRS duration (ms)	0.562 (0.374–0.749)	0.155	>166	37.50	78.00	0.494
Mean pulmonary valve area (mm^2^)	0.609 (0.438–0.779)	0.2249	>8.25	64.00	58.49	0.229
Pulmonary regurgitation fraction (%)	0.473 (0.284–0.661)	0.1311	>67.26	22.73	90.38	0.761

RS, peak radial strain; CS, peak circumferential strain; LS, peak longitudinal strain; RSRs, peak systolic radial strain rate; RSRe, peak early diastolic radial strain rate; CSRs, peak systolic circumferential strain rate; CSRe, peak early diastolic circumferential strain rate; LSRs, peak systolic longitudinal strain rate; LSRe, peak early diastolic longitudinal strain rate; LVEDVI, indexed LV end-diastolic volume; LVESVI, indexed LV end-systolic volume; RVEDVI, indexed RV end-diastolic volume; RVESVI, indexed RV end-systolic volume; LGE, late gadolinium enhancement.

**FIGURE 5:**
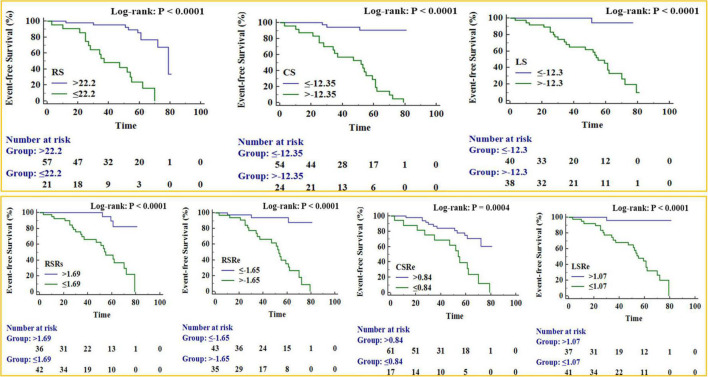
Kaplan–Meier survival curves for patient subgroups stratified by peak radial strain (RS), peak circumferential strain (CS), peak longitudinal strain (LS), peak systole radial strain rate (RSRs), peak early diastole radial strain rate (RSRe), peak circumferential strain rate at end-diastole (CSRe), and peak early diastole longitudinal strain rate (LSRe).

The Kaplan–Meier survival curves (Figure 5) verified that patients with significantly reduced RS, CS, LS, RSRs, RSRe, CSRe, and LSRe had worse event-free survival than those who did not have a significant reduction. The 5-year event-free cumulative survival rates were 68.6, 91, and 94% for patients with a preoperative RS of > 22.2% (*p* < 0.001), preoperative CS of < −12.35% (*p* < 0.001), and preoperative LS of < −12.3% (*p* < 0.001), respectively. As for the strain rate, the 5-year event-free cumulative survival rates were 74, 82.4, and 65.2% for patients with a preoperative RSRs of > 1.69 s^–1^ (*p* < 0.001), preoperative RSRe of < −1.65 s^–1^ (*p* < 0.001), and preoperative CSRe of ≥ 0.84 s^–1^ (*p* = 0.004), respectively. The median survival time was 62% for patients with a preoperative LSRe of > 1.07 s^–1^ (*p* < 0.001).

In univariate Cox analysis, both strain (RS, CS, and LS) and strain rate (RSRs, RSRe, CSRe, and LSRe) were significantly associated with adverse outcomes in patients with rToF after PVR (Table 4). The HRs of RS, CS, LS, RSRs, RSRe, CSRe, and LSRe were 0.845, 1.336, 1.342, 0.198, 2.450, 0.199, and 0.053, respectively. The C-indices of RS, CS, LS, RSRs, RSRe, CSRe, and LSRe were 0.811, 0.826, 0.801, 0.73, 0.624, 0.805, 0.75, 0.683, and 0.834, respectively. After multivariate correction, the predictive values of preoperative LS and LSRe remained statistically significant. Specifically, multivariate Cox regression analysis identified LS (HR = 1.243 [1.083–1.428]) and LSRe (HR = 0.067 [0.017–0.258]) as strong predictors of adverse events (Table 5).

**TABLE 4:** Univariate associations of clinical and cardiac magnetic resonance (CMR) characteristics with adverse events.

Predictors	Exp (B)	95.0% CI for Exp (B)		*P-value*
			
		Lower	Upper	
RVEF (%)	0.981	0.953	1.011	0.208
RVEDVI (ml/m^2^)	1.005	1.000	1.011	0.070
RVESVI (ml/m^2^)	1.011	1.003	1.019	0.006
RAVI (ml/m^2^)	1.010	0.958	1.035	0.136
RV LGE	1.625	0.870	3.846	0.145
LVEF (%)	0.979	0.953	1.005	0.109
LVEDVI (ml/m^2^)	1.017	1.003	1.031	0.014
LVESVI (ml/m^2^)	1.025	1.009	1.042	0.002
LAVI (ml/m^2^)	1.007	0.998	1.015	0.116
LV LGE	1.971	0.778	4.997	0.153
BSA (m^2^)	1.239	0.228	6.725	0.804
Heart rate beats (min)	1.022	0.99	1.055	0.174
QRSduration (ms)	1.006	0.994	1.018	0.366
Pulmonary regurgitation fraction (%)	1.013	0.991	1.035	0.262
Age at PVR (y)	1.023	0.989	1.058	0.181
Time interval between PVR and end point, y	0.827	0.77	0.888	<0.001
Age at surgical repair (y)	1.028	0.994	1.063	0.108
Time interval between rTOF and end point, y	1	0.998	1.002	0.843
mechanical valve	(−)	(−)	(−)	0.052
Bioprosthesis	2.919	0.971	8.774	0.056
Homograft	1.014	0.247	4.159	0.984
NYHA Class III or IV prior to PVR	3.829	1.711	8.572	0.001
Predicted RV pressure by TR (mmHg)	1.009	0.972	1.048	0.642
Servere tricuspid regurgitation	2.841	1.059	7.623	0.038
≥3previous cardiac operations	3.645	1.494	8.894	0.004
Pulmonary gradent at systolic (mmHg)	1.010	0.978	1.043	0.527
Presence of ventricular septum defect	0.619	0.230	1.662	0.341
Presence of associated anomalies	1.293	0.676	2.472	0.437
Related familial genetic syndromes	0.045	0	82.316	0.418
Sustained atrial arrhythmias before PVR	2.683	1.099	6.552	0.030
Sustained ventricular arrhythmias before PVR	/	/	/	/
LV mass/volume, g/ml	1.268	0.091	17.767	0.860
RV mass (g)	1.003	0.994	1.013	0.470
RV mass index (g/m^2^)	1.003	0.990	1.017	0.625
RV mass/volume (g/ml)	0.831	0.052	13.143	0.895
RV/LV end-diastolic volume ratio	1.040	0.577	1.873	0.897
RS (%)	0.845	0.732	0.911	<0.001
CS (%)	1.336	1.199	2.091	<0.001
LS (%)	1.342	1.065	1.612	<0.001
RSRs (s^–1^)	0.198	0.026	0.871	0.002
CSRs (s^–1^)	18.406	2.195	154.379	0.007
LSRs (s^–1^)	14.898	4.320	51.373	<0.001
RSRe (s^–1^)	2.450	1.659	3.618	<0.001
CSRe (s^–1^)	0.199	0.061	0.643	0.007
LSRe (s^–1^)	0.053	0.016	0.176	<0.001

CI, confidence intervals; RS, peak radial strain; CS, peak circumferential strain; LS, peak longitudinal strain; RSRs, peak systolic radial strain rate; RSRe, peak early diastolic radial strain rate; CSRs, peak systolic circumferential strain rate; CSRe, peak early diastolic circumferential strain rate; LSRs, peak systolic longitudinal strain rate; LSRe, peak early diastolic longitudinal strain rate; LVEDVI, indexed LV end-diastolic volume; LVESVI, indexed LV end-systolic volume; RVEDVI, indexed RV end-diastolic volume; RVESVI, indexed RV end-systolic volume.

**TABLE 5:** Multiple multivariable Cox to evaluate the associations of clinical and cardiac magnetic resonance (CMR) characteristics with adverse events.

		95.0% CI		
	Exp(B)	for Exp(B)		*P-value*
			
		Lower	Upper	
RS (%)				
CS (%)				
LS (%)	1.243	1.083	1.428	0.002
RSRs (s^–1^)				
RSRe (s^–1^)				
CSRe (s^–1^)				
LSRe (s^–1^)	0.067	0.017	0.258	<0.001

LSRe (s^–1^)	0.067	0.017	0.258	<0.001
LS (%)	1.243	1.083	1.428	0.002
LVEDVI (ml/m^2^)				
LVESVI (ml/m^2^)				
RVEDVI (ml/m^2^)				
RVESVI (ml/m^2^)				

LSRe (s^–1^)	0.067	0.017	0.258	<0.001
LS (%)	1.243	1.083	1.428	0.002
RV mass-ED (g)				
RV mass index (g/m^2^)				
RV mass/volume (g/ml)				
RV/LV end-diastolic volume ratio				

LSRe (s^–1^)	0.083	0.016	0.437	0.003
LS (%)	1.274	1.041	1.561	0.019
RVOT area (cm^2^)				
Pulmonary regurgitation fraction (%)				
Predicted RV pressure by TR (mmHg)				
Severe tricuspid regurgitation				

LSRe (s^–1^)	0.066	0.017	0.262	<0.001
LS (%)	1.256	1.089	1.447	0.002
Age at surgical repair (years)				
Age at PVR (years)				
≥3previous cardiac operations				
Related familial genetic syndromes				
NYHA Class III or IV prior to PVR				

LSRe (s^–1^)	0.093	0.023	0.371	0.001
LS (%)	1.29	1.098	1.515	0.002
QRS duration (ms)				
Heart rate (beats/min)				
Sustained atrial arrhythmias prior to PVR				

Multivariate analysis Cox regression (forward LR) was carried out for those parameters who were confirmed statistically significant in univariate analysis and important function and clinical parameters. CI, confidence intervals; RS, peak radial strain; CS, peak circumferential strain; LS, peak longitudinal strain; RSRs, peak systolic radial strain rate; RSRe, peak early diastolic radial strain rate; CSRe, peak early diastolic circumferential strain rate; LSRe, peak early diastolic longitudinal strain rate; LVEDVI, indexed LV end-diastolic volume; LVESVI, indexed LV end-systolic volume; RVEDVI, indexed RV end-diastolic volume; RVESVI, indexed RV end-systolic volume.

Regarding cardiac function parameters, in the univariate Cox analysis, RVESVI, LVEDVI, and LVESVI were associated with worse outcomes after PVR, whereas RVEDVI, RVEF, and LVEF were not. In addition to NYHA class III or IV prior to PVR, severe tricuspid regurgitation, ≥ 3 previous cardiac surgeries, sustained atrial arrhythmias prior to PVR, and the time interval between PVR and endpoint were related to adverse events after PVR (Table 4). However, after multiple multivariate corrections, only the predictive values of preoperative LS and LSRe remained significant (Table 5).

### Intra- and inter-observer reliability

In a subgroup of 20 patients, reliability of strain was generally high with an intra-observer difference of approximately 0.01 ± 0.28%–0.12 ± 0.26%, and an inter-observer difference of approximately 0.01 ± 0.24%–0.15 ± 0.31%. Similarly, intra- and inter-observer differences were also small for strain rates (approximately 0.01 ± 0.35–0.13 ± 0.24 s^–1^ and 0.01 ± 0.29–0.10 ± 0.30 s^–1^, respectively; Bland–Altman plots are presented in Supplementary Figures 1, 2).

## Discussion

In the present study, we found the following findings: (1) factors, including conventional volume parameters of both ventricles, ≥ 3 previous cardiac surgeries, severe TR, sustained atrial arrhythmias prior to PVR, and NYHA III/IV, were all associated with outcomes of PVR in patients with rToF; (2) preoperative LV RS, CS, LS, RSRs, CSRe, RSRe, and LSRe were significantly associated with adverse outcomes after PVR surgery in patients with rToF, suggesting that they serve as a novel index for risk stratification assessment in patients with rToF after PVR; and (3) multiple variable analysis demonstrated that patients with the highest incidence of adverse events were those with a severe reduction in LV LS and/or LSRe prior to PVR surgery.

Progressive PR is one of the most common complications occurring after rToF, despite the current treatment strategies used to treat ToF, demonstrating long-term survival (30-year survival rate between 68.5 and 90.5%) (25). In response to longstanding regurgitation, the RV progressively dilates to adapt to the increasing load (26), and the patients’ exercise tolerance progressively reduces. In such situations, PVR is an effective method to help restore pulmonary valve function and improve cardiac function while alleviating symptoms (27). Our study confirmed for the first time that a severe decrease in RS, CS, LS, RSRs, RSRe, and LSRe was associated with an increase in the incidence of adverse outcomes during a midterm follow-up. Among these, LS and LSRe before PVR were the strongest risk factors for postoperative outcomes. After adjusting for other clinical and cardiac function parameters, these strain parameters maintained their prognostic values. This was supported by several CMR feature tracking-derived myocardial function indices, in which LSRe was utilized in the assessment of LV diastolic function and proved to be the most stable parameter with high reproducibility at both intra- and inter-observer levels (28).

Several factors influence the prognosis of PVR, most of which are clinical indicators or surgery-related factors. Jang et al. (29) conducted a retrospective study of 131 PVRs to explore the midterm clinical results of PVR after rToF. In univariate analysis, they found that the type of valve implanted, large valve implantation, and young age for rToF (<15 years) were risk factors for repeated PVR. Dorobantu et al. found that PVR after 35 years of age was associated with worse outcomes (30). In our study, we ascertained that the age for surgical repair of the ToF and PVR was significantly higher in patients with no events than in those with events; however, this difference did not affect the outcomes after PVR. Moreover, Jang et al. (29) found that the type of implanted valve and valve size were risk factors, whereas our study did not. The use of only biological valves may be the culprit, whereas, in our study, the patients used mechanical valves as well as homogenous valves, in addition to biological valves. We did not record this data in terms of valve size.

Furthermore, in our study, the pre-PVR LV and RVEF were not associated with adverse events, whereas previous studies, including the INDICTOR study (31), found that RVEF has a predictive value for mortality (*P* = 0.03). The reason may be that the RVEF values of our study, in both the event and no event groups, were relatively low (<40%), and our study had a shorter average follow-up time (3.6 vs. 9.5 years). Similar to the INDICTOR study, we found that LVESVI and atrial arrhythmias prior to PVR are related to prognosis; however, we did not find the predicted value of the RV mass-to-volume ratio and RV systolic blood pressure, as mentioned in their study. In addition, we found that LVEDVI and RVESVI were correlated with prognosis, which is consistent with the findings of other studies. However, after incorporating the strain parameter into the multivariate analysis, the predictive value of these parameters ceased to exist.

Moreover, Roderick et al. found that prolonged QRS duration prior to PVR is the main determinant of the poor long-term follow-up outcome of patients with ToF (32); however, our study did not find such a result. Some studies found that VO^2^ peak (%), which assesses the degree of exercise tolerance, may be a useful predictor of adverse events after PVR (33, 34). Although we were not able to obtain the results of the cardiopulmonary exercise experiment, we also concluded that patients with NYHA III/IV have a poor prognosis based on the NYHA classification, which correlates with the study conducted by Anna et al. (35). Anna et al. also found that ≥ 3 previous cardiac surgeries were associated with worse outcomes, which is consistent with the findings of our study. Their study also found that older age at rToF and greater BSA at PVR were factors influencing poor prognosis; however, this phenomenon was not observed in our study.

One study demonstrated that in multivariate analysis, severe preoperative TR (HR = 2.49; 95% confidence interval [CI], 1.11–5.52), right ventricular end-systolic volume (HR = 1.02; 95% CI, 1.01–1.03), and age at PVR can predict adverse events (36). However, in our study, the value of severe preoperative TR and right ventricular end-systolic volume to predict adverse events was only found in the univariate analysis. In the multivariate analysis, these parameters were not useful. Age at PVR was not significantly different between univariate and multivariate analyses.

This study had some limitations that must be highlighted. For instance, our study was conducted in one medical center with a relatively small sample size; thus, in addition to GLS, the feasibility of other parameters, such as GCS, should be further explored. Large-sample multicenter studies with possible confounders are required to validate and improve the power of assessing events. Another limitation of the study is the cardiac endpoint utilized, which includes a wide range of heterogeneous events from hard to softer ones. Moreover, it was determined that the number of outcomes was modest, although this is the largest study of patients with rToF after PVR (31). The number of hard events was small because the sample size was small, and contrastingly, partly because the cardiac function of such patients significantly improved after PVR (13). In addition, due to limited research conditions, we did not report inter-study reproducibility. However, inter-study reproducibility of strain measured by CMR feature tracking has been reported in a previous study, showing a satisfactory coefficient of variation and intraclass correlation coefficient (37). Lastly, it was shown that significant differences were noted in all strain and strain rate values between different vendors. Because we only provided the strain and strain rate data of the Circle CVI software (38), the data of other vendors, such as TomTec Arena, QStrain Medis, and Segment Medviso, still need to be further supplemented by other studies.

In conclusion, PVR in patients with rToF has relatively low mortality and fewer adverse events. Preoperative LV RS, CS, LS, RSRs, RSRe, CSRe, and LSRe were significantly associated with adverse outcomes after PVR. In particular, LS and LSRe assessed with CMR are independent predictors of survival in patients with rToF after PVR and offer incremental information for risk stratification beyond clinical parameters, biomarkers, and standard CMR parameters.

## Data availability statement

The raw data supporting the conclusions of this article will be made available by the authors, without undue reservation.

## Ethics statement

The studies involving human participants were reviewed and approved by Fuwai Hospital Ethics Department. Written informed consent to participate in this study was provided by the participants or their legal guardian/next of kin.

## Author contributions

SZ, SL, and ML: guarantors of integrity of entire study. BZ, SY, ZF, and ML: literature research. BZ, SY, FH, and YJ: clinical studies. BZ and ML: statistical analysis. BZ, SZ, SL, and ML: manuscript editing. All authors have study concepts/study design or data acquisition or data analysis/interpretation, manuscript drafting or manuscript revision for important intellectual content, approval of final version of submitted manuscript, and agreed to ensure any questions related to the work are appropriately resolved.

## Supplementary material

Supplementary Figure 1 Bland–Altman analysis for intra-observer reproducibility of strain and strain rate (the blue line indicates the mean value; the dashed red line indicates 95% CI).

Supplementary Figure 2 Bland–Altman analysis for inter-observer reproducibility of strain and strain rate (the blue line indicates the mean value; the dashed red line indicates 95% CI).
