# Supplementary figures and images for: Left ventricular strain derived from cardiac magnetic resonance can predict outcomes of pulmonary valve replacement in patients with repaired tetralogy of Fallot
Source: Front Cardiovasc Med. 2022 Aug 18;9:917026. doi: 10.3389/fcvm.2022.917026 (PMC9433663; doi:10.3389/fcvm.2022.917026)

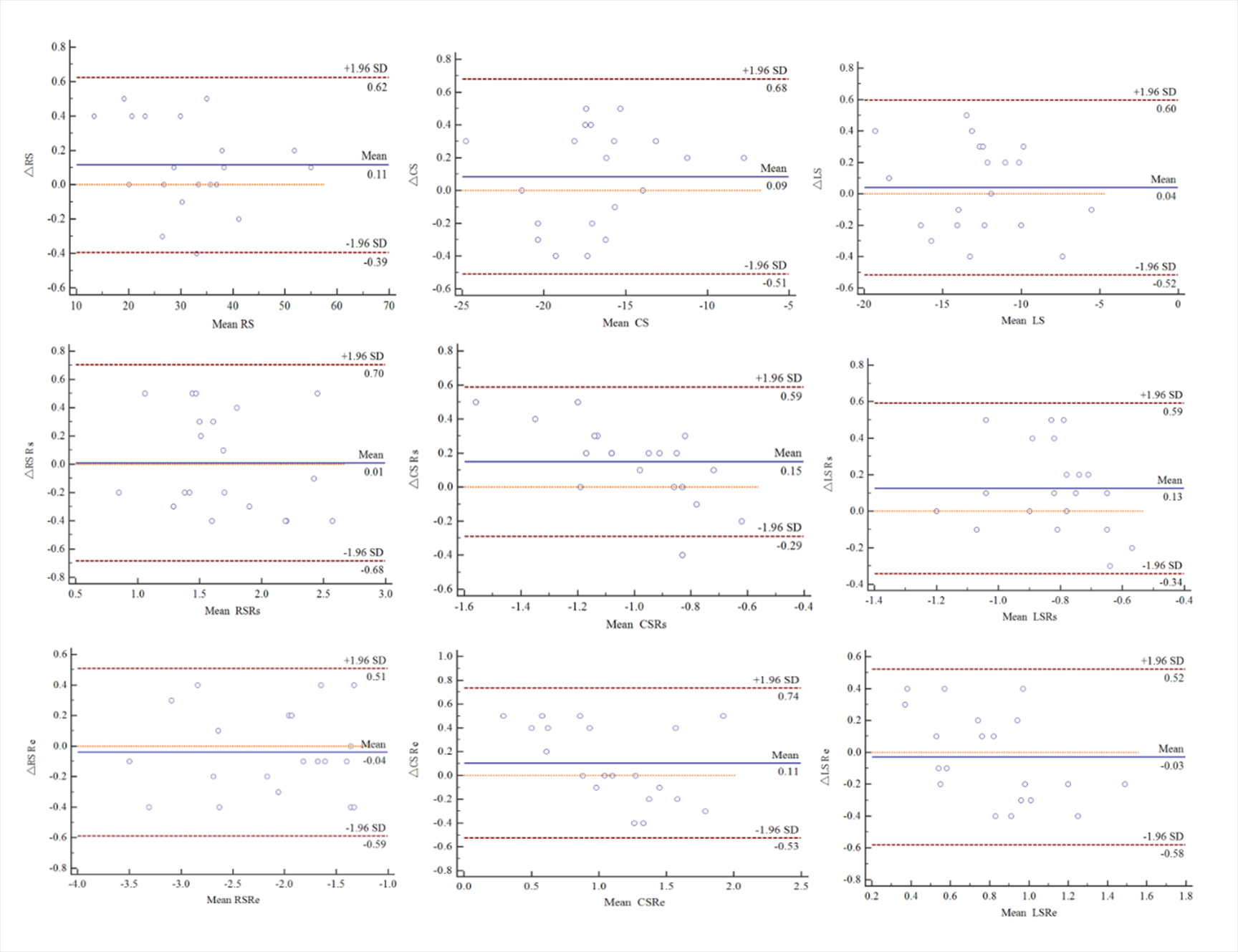

Supplement: Supplementary Figure 1 — Bland–Altman analysis for intra-observer reproducibility of strain and strain rate (the blue line indicates the mean value; the dashed red line indicates 95% CI). [file Image_1.tif]

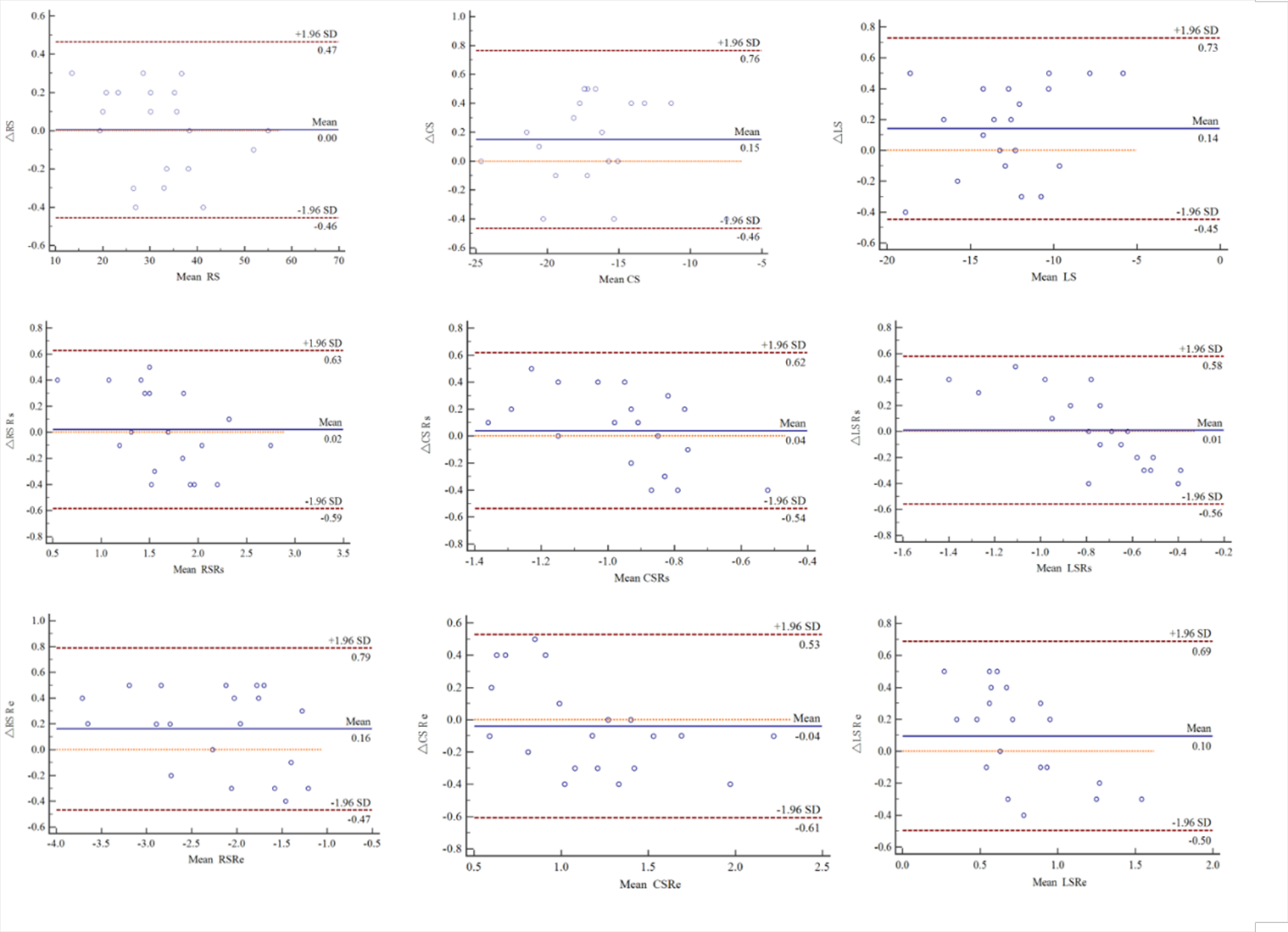

Supplement: Supplementary Figure 2 — Bland–Altman analysis for inter-observer reproducibility of strain and strain rate (the blue line indicates the mean value; the dashed red line indicates 95% CI). [file Image_2.tif]
